# Intracellular Acid-Extruding Regulators and the Effect of Lipopolysaccharide in Cultured Human Renal Artery Smooth Muscle Cells

**DOI:** 10.1371/journal.pone.0090273

**Published:** 2014-02-21

**Authors:** Shih-Hurng Loh, Chung-Yi Lee, Yi-Ting Tsai, Shou-Jou Shih, Li-Wei Chen, Tzu-Hurng Cheng, Chung-Yi Chang, Chein-Sung Tsai

**Affiliations:** 1 Department of Pharmacology, National Defense Medical Center, Taipei City, Taiwan; 2 Department of Cardiovascular Surgery, Tri-Service General Hospital, Taipei, Taiwan; 3 Department of Biological Science and Technology, College of Life Sciences, China Medical University, Taichung, Taiwan; 4 Department of General Surgery, Cheng-Hsieng General Hospital, Taipei, Taiwan; University of Szeged, Hungary

## Abstract

Homeostasis of the intracellular pH (pH_i_) in mammalian cells plays a pivotal role in maintaining cell function. Thus far, the housekeeping Na^+^-H^+^ exchanger (NHE) and the Na^+^-HCO_3_^−^ co-transporter (NBC) have been confirmed in many mammalian cells as major acid extruders. However, the role of acid-extruding regulators in human renal artery smooth muscle cells (HRASMCs) remains unclear. It has been demonstrated that lipopolysaccharide (LPS)-induced vascular occlusion is associated with the apoptosis, activating calpain and increased [Ca^2+^]_i_ that are related to NHE1 activity in endothelia cells. This study determines the acid-extruding mechanisms and the effect of LPS on the resting pH_i_ and active acid extruders in cultured HRASMCs. The mechanism of pH_i_ recovery from intracellular acidosis (induced by NH_4_Cl-prepulse) is determined using BCECF-fluorescence in cultured HRASMCs. It is seen that (a) the resting pH_i_ is 7.19±0.03 and 7.10±0.02 for HEPES- and CO_2_/HCO_3_^−^- buffered solution, respectively; (b) apart from the housekeeping NHE1, another Na^+^-coupled HCO_3_^−^ transporter i.e. NBC, functionally co-exists to achieve acid-equivalent extrusion; (c) three different isoforms of NBC: NBCn1 (SLC4A7; electroneutral), NBCe1 (SLC4A4; electrogenic) and NBCe2 (SLC4A5), are detected in protein/mRNA level; and (d) pH_i_ and NHE protein expression/activity are significantly increased by LPS, in both a dose- and time- dependent manner, but NBCs protein expression is not. In conclusion, it is demonstrated, for the first time, that four pH_i_ acid-extruding regulators: NHE1, NBCn1, NBCe1 and NBCe2, co-exist in cultured HRASMCs. LPS also increases cellular growth, pH_i_ and NHE in a dose- and time-dependent manner.

## Introduction

Atherosclerotic renal artery stenosis (ARAS) is the most common primary disease of the renal arteries and it is associated with hypertension [1], [2]. In addition to threatening renal function, ARAS-induced atherosclerotic renovascular disease with renal failure and can result in mortality [3]–[6]. Many important cellular functions are affected by a change in pH_i_. Mechanisms such as cell volume [7], the permeability of ion channels [8], enzyme catalysts [9], cell differentiation, growth and apoptosis are all sensitive to changes in pH_i_ [10]–[12]. These disturbances in pH_i_ have also recently been claimed to be responsible for the development of hypertension and vascular atherosclerosis in animal models [12], [13]. However, the evaluation of the biological effect on the cell of acid-base transport in vascular cells is difficult, especially in human tissues/cells.

The pH_i_ in mammalian cells remains within a narrow range (7.0–7.2) because of the combined operation of active transmembrane transporters and passive intracellular buffering power [14]. The membrane transporters can be divided into two main categories: acid extrusion carriers and acid loading carriers. Acid extrusion carriers, such as Na^+^/H^+^ exchanger (NHE) and Na^+^/HCO_3_^−^ cotransporter (NBC), are activated when cells are become acidic (pH_i_ <7.1) [12], [15], [16]. Net acid extrusion from vascular smooth muscle cells (VSMCs) in rat and mice mesenteric small arteries is mediated by the Na^+^-HCO_3_^−^-cotransporter NBCn1 (slc4a7) and the Na^+^/H^+^-exchanger NHE1 (slc9a1) [12], [15], [17], [18]. NHE mediates the electroneutral exchange of extracellular Na^+^ for intracellular H^+^ [12], [19], [20]. pH_i_ recovery in HEPES buffered media (HCO_3_^−^-free condition) is inhibited by the removal of extracellular Na^+^ or by the addition of amiloride or Hoe 694 (3-methylsulfonyl-4-piperidinobenzoyl, guanidine hydrochloride), a compound that inhibits NHE activity because of its high affinity and selectivity [21]. NHE proteins comprise a family of at least ten NHE members (NHE 1–9 and sperm NHE), each of which is expressed by a separate gene [22], [23]. Different subtypes of NHE occur in different cell types. In rat/mouse VSMCs, the transporter is well defined at the molecular level as NHE1 [12], [18], [24].

Na^+^-HCO_3_^−^-dependent transporter is largely, 4-diisothiocyanatostilbene-2,2- disulphonic acid (DIDS)-sensitive (56% to 91%), and it is amiloride- and HOE 694-resistant [12], [16], [25]–[28]. It is also inhibited by the removal of external Na^+^ [29]. Relevant molecular candidates for Na^+^-dependent bicarbonate transport include at least five members of the slc4 family [12], [27], [30]. It has been recently found that NBCn1 (SLC4A7) mediates the Na^+^-dependent bicarbonate transport that is important for acid extrusion in the smooth muscle cells of mouse mesenteric, coronary, and cerebral small arteries [12], [15], [17], [27], [31]. However, thus far there have been no related reports about active acid extruding transports in human renal artery smooth muscle cells (HRASMCs).

Lipopolysaccharides (LPS) of gram-negative bacteria play a pivotal role in septic shock syndrome in humans [32]. It has been demonstrated that mRNA and protein expression of toll-like receptor 4 (TLR4) are up-regulated by LPS in human aortic smooth muscles, in a dose- (10∼1000 ng/ml) and time-dependent (0–48 hr) manner [33]. In human arterial smooth muscle, LPS (10 ng/ml) has also been found to induce mRNA and protein expression of matrix metalloproteinases-9 (MMP-9), in a TLR4/NF-kB-dependent manner [34]. Previous studies have shown that the inhibition of NHE1 has anti-apoptotic effects [35]–[37]. A very recent study in HUVECs has further demonstrated that LPS increases NHE1 activity, in a time-dependent manner that is associated with an increased [Ca^2+^]_i_, which results in enhanced calpain activity and apoptosis, via NHE1-dependent degradation of Bcl-2 [38]. However, thus far, the effect of LPS on resting pH_i_ and acid-extruding regulators has not been determined in HRASMCs. In this study, apart from checking the resting pH_i_ and the acid extruding regulators in HRASMCs, the dose- (1–10000 ng/ml) and time- (6∼48 hrs) dependent effect of LPS on resting pH_i_ and the possible acid extruders is also studied.

In brief, this study demonstrates, for the first time, that two different types of acid-extruders: NHE1 and NBC, functionally co-exist in cultured HRASMCs. Three different isoforms of Na^+^-coupled HCO_3_^−^ co-transporter: NBCn1 (SLC4A7; electroneutral), NBCe1 (SLC4A4; electrogenic) and NBCe2 (SLC4A5), are detected in protein/mRNA level. It is also demonstrated that LPS increases cellular growth, pH_i_ and NHE in a dose- and time-dependent manner, but does not affect the activity of NBCs.

## Materials and Methods

### Human renal artery smooth muscle cells (HRASMCs)

With the approval of the Institutional Review Board of Tri-Service General Hospital, National Defense Medical Center (TSGHIRB No. 1-101-05-065) and with prior written informed consent of patients, 9 renal artery segments (5 male: 62.5±6.3 yrs old, 4 female: 60.6±7.2 yrs old) were collected from surgically-leftover specimens of human renal arteries during renal transplant surgery at Tri-service General Hospital, Taipei, Taiwan. Primary HRASMCs were isolated by the explant technique which has been described in detail in Fletcher et al [39] and cultured in HAM's F12K medium containing 10% fetal bovine serum (FBS) (GIBCO, Grand Island, NY, U.S.A.), 100 U/ml penicillin, 100 mg/ml streptomycin, and 200 mM L-glutamate in a humidified incubator (at 37°C and 5% CO_2_). The primary HRASMCs were used for experiments between 3 and 8 passages. The preparations were then perfused with oxygenated Tyrode solution, which was either 100% O_2_ for nominally bicarbonate-free Tyrode solution or 5% CO_2_/95% O_2_ for bicarbonate-containing Tyrode solution, at 37°C, pH 7.40±0.02 for experiments.

### Immunocytochemistry

Cells were cultured on a 6-well plate (Macalaster Bicknell, New Haven, CT) for 1–3 days. Cells were subsequently washed twice in phosphate-buffered saline (PBS). After washing, cells were fixed in 4% paraformaldehyde for 30 min at room temperature, then washed twice in PBS and blocked and permeabilized in PBS containing 0.3% triton and 5% normal goat serum for 60 min, and finally washed in PBS and incubated overnight with primary antibodies at 4°C. Cells were washed four times in PBS and labeled with secondary antibodies for 1 h in the dark. After labeling, cells were washed with PBS and incubated with DAPI (1: 200) for 40 min. Cells were washed twice more and mounted onto slides with Gel/Mount (Biomedia Corp., Forest City, CA). Images were acquired with an OLYMPUS 200M (Japan) microscope system.

### Western blot analysis of the NHE-1∼3 protein and SLC4 family of HCO_3_^−^ transporters

Cell lysates were prepared using a 300 μl/well of six-well plates of RIPA/NP-40 lysis buffer (5 mM Tris pH 7.4, 30 mM NaCl, 1 mM PMSF, 1 μg/ml aprotinin). A 50 μg of total protein per sample was then subjected to 10% PAGE, transferred to a PVDF membrane and blocked for 2 h with 5% fat-free milk in Tris-buffered saline/0.1% Tween 20 (TBST). After three washes with TBST, the membranes were exposed to a 1: 1000 dilution of a mouse antihuman NHE1∼3 antibody (Millipore, Long Beach, CA, USA) [40] or SLC4 family of HCO_3_^−^ transporters:NBCe1 (Millipore, Long Beach, CA, USA) [41], [42], NBCe2 (Abgent, San Diego, CA, USA), NBCn1 (Abgent, San Diego, CA, USA) and NDCBE (GeneTex, San Antonio, TX) at 4°C overnight. Following three washes with TBST, the membrane was exposed to a 1: 15000 dilution of goat anti-mouse IgG-HRP conjugate (Millipore, Long Beach, CA, USA) for 1 h and washed repeatedly with TBST. Chemiluminescence was detected using the SuperSignal Substrate (PIERCE, Rockford, IL, USA). Loading control was assessed by the detection of β-actin. NHE-1∼3, SLC4 family of HCO_3_^−^ transporters: SLC4A4 (NBCe1), SLC4A5 (NBCe2), SLC4A7 (NBCn1), SLC4A8 (NDCBE) and β-actin protein intensity were measured using the Analytical Imaging Station software version 2. The specificity of the applied antibodies can be checked in the quoted reference(s) or the datasheet of the company. Briefly, the specificity of the NBC antibodies used in this study are shown as following: NBCe1 is recognized by a major band of approximately 130 kDa and a major band of approximately 160 kDa in salamander kidney; NBCe2 is generated from rabbits immunized with a KLH conjugated synthetic peptide between 1073–1102 amino acids from the C-terminal region; NBCn1 is generated from rabbits immunized with a KLH conjugated synthetic peptide between 1193–1222 amino acids from the c-terminal region; NDCBE is generated from rabbits and recognized by a major band of 123 kDa.

### Reverse Transcription – Polymerase Chain Reaction (RT-PCR)

Total RNA was isolated using GeneJET RNA Purification Kit (Thermo Scientific, MA, USA) from human renal artery smooth muscle cells (HRASMCs). The RNA (1 μg) was reverse transcribed using Maxima First Strand cDNA Synthesis Kit (Thermo Scientific, MA, USA). PCR (DreamTaq Master Mix, Thermo Scientific, MA, USA) with 10∼50 ng cDNA and 1 pmol of each primer (NBCe1-SLC4A4: forward: 5-GGTGTGCAGTTCATGGATCGTC-3; reverse: 5′-GTCACTGTCCA- GACTTCCCTTC-3′; NBCe2-SLC4A5: forward: 5′-ATCTTCATGGACCAGCA- GATCAC-3′; reverse: 5′-TGCTTGGCTGGCATCAGGAAG-3′; NBCn1- SLC4A7: forward: 5′-CAGATGCAAGCAGCCTTGTGTG-3′; reverse: 5′-GGTCCATGATG- ACCACAAGCTG-3′; NDCBE1-SLC4A8:forward: 5′-GCTCAAGAAAGGCTG- TGGCTAC-3′; reverse: 5′-CATGAAGACTGA GCAGCCCATC -3′; Actin: forward: 5′-AGAAGAGCTACGAGCTGCCTG-3′; reverse: 5′-CTCCTGCTTGC- TGATCCACATC-3) was performed for 30 cycles after 15 min at 95°C: 95°C, after which denaturation was performed for 30 s at 60°C, annealing for 30 s, and elongation at 72°C for 1 min. Negative controls included omission of cDNA. PCR for actin was performed to validate each template. PCR products were separated by 2% agarose gel electrophoresis with ethidium bromide and photographed under ultraviolet illumination. All primer pairs were confirmed by nucleotide sequencing representative PCR products.

### MTT assay

Cells were seeded onto 24-well plates at a density of 3×10^4^ cells/well and grown for up to 24 hr with 1 ml serum free medium. The growth medium was replaced on day 2 with a different dosage of LPS (1∼10000 ng/ml) for another 24 hr. Cell viability was assessed by using the 3-(4, 5-dimethyl-2-thiazolyl)-2, 5-diphenyl-2H-tetrazolium bromide (MTT) assay (Sigma) according to the manufacturer's protocol. In brief, an ELISA (Enzyme-linked immunosorbent assay) reader was used to detect the absorbance of 490 nm after 10% MTT had been reacted with different tested cells for 3 hr.

### Measurement and calibration of the intracellular pH

**Figure 1:**
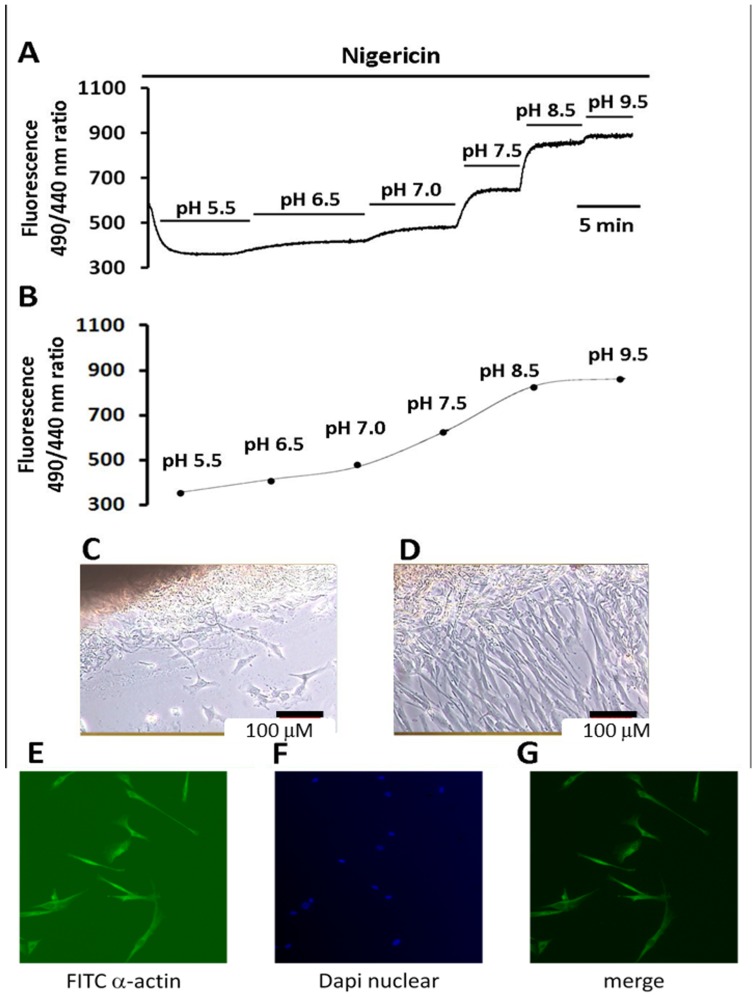
In situ calibration of intracellular pH, purity and identification of human renal artery smooth muscle cells. **A & B**: In situ intracellular pH calibration curve in human renal artery smooth muscles cells (HRASMCs). **A**: The trace shows the BCECF fluorescence (510 nm emission at 490 nm and 440 nm excitations) in HRASMCs. (Please see *Materials and Methods* for details). **B**: The curve shows data pooled from 6 similar experiments shown in A. **C & D**: Phase-contrast micrographs of cultured HRASMCs (10×40), using explant technique. Cell cultured at the 10th day (**C**) and 20 days (**D**). The dark black area at the left top corner is the renal artery tissue. The bar below represents a length of 100 μm. **E, F & G**: Micrographs of immunohistochemistry of HRASMCs. **E**: HRASMCs stained for the anti-smooth muscle alpha actin (green). **F**: HRASMCs counterstained with DAPI for nuclei (blue). **G**: A merge micrograph that combines micrograph E and micrograph F (10×40).

Measurement of the pH_i_ has been described in detail in our previous reports [16], [26]. In brief, the pH_i_ in the cultured HRASMC was measured using the pH-sensitive, dual excitation single-emission fluorescent dye, 2′,7′-bis(2-carboxethyl)-5(6)-carboxy-fluorescein–acetoxymethyl (BCECF-AM) (Molecular Probes). The preparations were loaded with BCECF-AM (5 μM) by incubating them for 30 min at room temperature and exciting them alternately with 490 and 440 nm wavelength light. The BCECF fluorescence emission ratio of the 510 nm emission at 490 nm and 440 nm excitation (490/440) was calibrated using the K^+^-nigericin method [16]. Briefly, this method consisted of exposing a BCECF-loaded cell to the six nigericin calibration solutions (listed below in the *Solution* section) that clamps pH_i_ to the value of pH_o_ of the calibration solution. Fig. 1A showed the emission ratio changes seen on perfusing human artery smooth muscle cells with calibration solutions with different 6 pH values (5.5∼9.5) in the presence of 10 μM nigericin. The emitted ratio 510 nm emission at 490 nm and 440 nm excitations (R; R = F_490_/F_440_) was increased as the pH value of superfusing solution was increased. R_max_ and R_min_ are, respectively, the maximum and minimum ratio values for the data curve. The fluorescence of BCECF at 490 nm to 440 nm is a function of pH_i_ and the overall sampling rate in the experiment was 0.5 Hz for the recorded fluorescent ratio (490 nm/440 nm). Using the linear regression fit of the data (shown in the Fig. 1B) obtained from 6 calibration experiments similar to that shown in Fig. 1A, the mean apparent dissociation constant (pKa) at 37°C was found to be 7.18, very close to the value determined by our previous study of the human heart, as well as the value determined by other investigators [16], [43], [44]. The following equation [45] was used to convert the fluorescent ratio in to pH_i_:
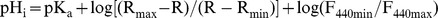
where R is the ratio of the 510 nm fluorescence at 490 nm and 440 nm excitation, R_max_ and R_min_ are, respectively, the maximum and minimum ratio values from the data curve and the pK_a_ (-log of dissociation constant) is 7.18. F_490min_/F_440min_ and F_490max_/F_440max_ is the ratio of fluorescence measured at 440 nm of R_min_ and R_max_, respectively.

### Experimental alteration of intracellular pH- weak acid/base pre-pulse technique

**Figure 2:**
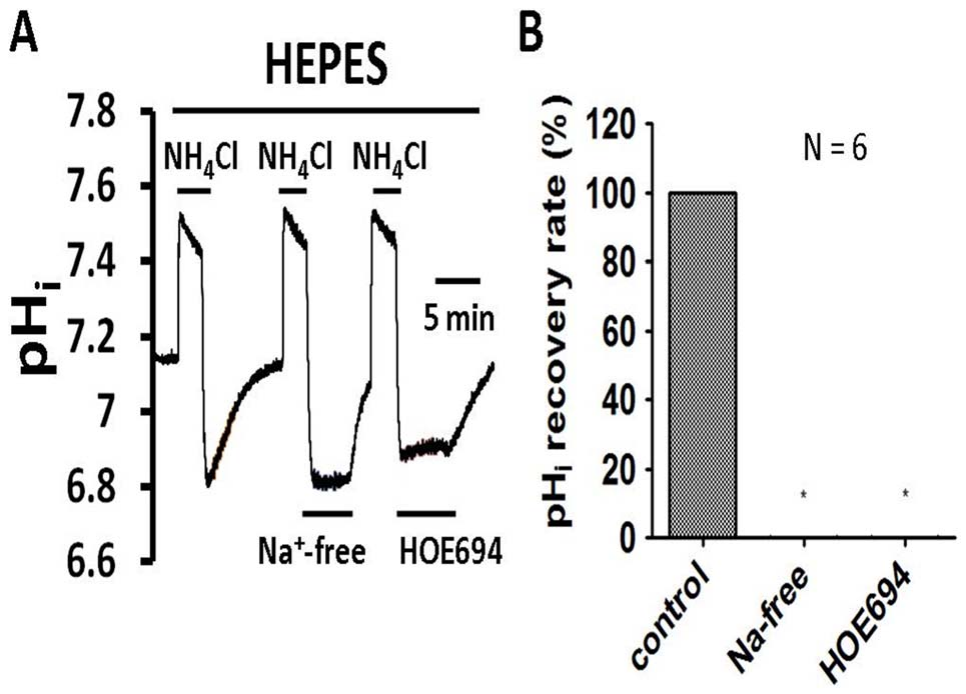
Effect of Na^+^-free and 30 μM HOE 694 on pHi recovery from induced acidosis (evidence of Na^+^-H^+^ exchanger) in HRASMCs superfused with HEPES-buffered Tyrode solution. **A:** Top bar shows buffer system used in the superfusate. The periods of application of NH_4_Cl and tested drugs (30 μM HOE 694, a NHE exchanger inhibitor, and Na^+^-free solution) are indicated with bars above or below the trace. The left part of trace A show a typical recovery of pH_i_-recovery from an intracellular acidosis induced by a 10 min NH_4_Cl (20 mM) pre-pulse in HEPES-buffered Tyrode solution (pHo  = 7.4, 37°C) in HRASMCs. For details of the mechanism of the pre-pulse technique, please see the *Materials and Methods* section. The right part of trace A represents experiments showing the effect of Na^+^-free and 30 μM HOE 694 on pH_i_ recovery, respectively, in HRASMCs. **B:** Histograms, showing the pH_i_ recovery slope of acid extrusion after NH_4_Cl-induced intracellular acidosis averaged for 6 experiments similar to those shown in A. *: p<0.01 vs. control.

NH_4_Cl pre-pulse techniques were used in the present work to induce acute acid loading [44], [46]. NH_4_Cl pre-pulses were achieved with (∼10 minute) extracellular exposures to 20 mM NH_4_Cl. Briefly, the mechanism of the NH_4_Cl prepulse technique relies upon the characteristic of incomplete dissociation. For example, the details of NH_4_Cl prepulsing procedures, used in the present study, are given below. It can be explained in terms of four phases as shown in Fig. 2: rapid entry (see phase 1 in left part of Fig. 2A), slow recovery (see phase 2 in left part of Fig. 2A), rapid exit (see phase 3 in left part of Fig. 2A), and pH_i_ regulation (see phase 4 in left part of Fig. 2A) that the sudden acidosis activates pH_i_ regulatory proteins in the membrane, for instance Na^+^-H^+^ exchanger and Na^+^-HCO_3_^−^ cotransporter. Throughout the whole experiment, the change of pH_i_ induced by the tested drug was compared around the 3^rd^ min after treating the drug (the testing pH_i_ is around 6.90±0.15), unless otherwise stated. The background fluorescence and auto-fluorescence were small (<5%) and haves been ignored.

### Chemicals and solutions

*Standard HEPES-buffered Tyrode solution* (air equilibrated) contained (mM): NaCl, 140; KCl, 4.5; MgCl_2_, 1; CaCl_2_ 2.5; glucose, 11; HEPES, 20; pH adjusted to 7.4 with 4N NaOH. Unless otherwise stated, pH adjustments of all HEPES-buffered solutions were performed at 37°C (these adjustments included those where ionic-substitutions were made, see below). *Standard bicarbonate-buffered Tyrode solution* (equilibrated with 5% CO_2_/23 mM HCO_3_^−^) was the same as above, except that the sodium chloride concentration was reduced to 117 mM, and 23 mM NaHCO_3_ was added instead of the HEPES (pH 7.40 at 37°C).

*Ion-substituted solutions:* In a *Na^+^-free, HEPES-buffered Tyrode solution*, (air equilibrated) contained (mM): N-methly-D-glucamine (NMDG), 140; KCl, 4.5; MgCl_2_, 1; CaCl_2_ 2.5; glucose, 11; HEPES, 20, and the pH was adjusted to 7.4 with HCl. The *Na^+^-free CO_2_/HCO_3_^−^-buffered Tyrode solution* (equilibrated with 5% CO_2_/23 mM HCO_3_^−^) contained (in mM): NMDG, 140; KCl, 4.5; CaCl_2_ 2.5; MgCl_2_, 1; glucose, 11, and pH was adjusted to 7.4 at 37°C with HCl under the condition of saturating with 5% CO_2_/95% O_2_. When 20 mM ammonium chloride was used, it was added directly as a solid to solution without osmotic compensation. 4,4′-diisothiocyanatostilbene-2,2′-disulphonic acid (DIDS) and HOE 694 (3-methylsulfonyl-4-piperidinobenzoyl, guanidine hydrochloride) were added, as solids, to solutions shortly before use.

*Nigericin calibration solutions* contained (mM): KCl, 140; MgCl_2_, 1; 10 μM nigericin; buffered with one of the following organic buffers: 20 mM 2-(N-morpholino) ethanesulphonic acid (MES, pH 5.5), 20 mM HEPES (pH 7.5) or 20 mM 3-(cyclohexylamino)-2-hydroxy-1-propane-sulphonic acid (CAPSO, pH 9.5), and were adjusted (37°C) to the correct pH with 4N NaOH.

HOE 694 was kindly provided by Hoechst Aktiengesellshaft (Germany). All other chemicals were from Sigma (UK) and Merck (UK).

### Statistics

All data are expressed as the mean ± the standard error of the mean (SEM) for n preparations. Statistical analysis was performed using one-way analysis of variance (one-way ANOVA) with Scheffe's posterior comparison. A P value smaller than 0.05 was regarded as significant.

## Results

### The isolation of human renal artery smooth muscle cells (HRASMCs) from tissue

This study successfully isolated HRASMCs from human artery tissue, using the so-called explant method. The HRASMs were significantly migrate out of the artery tissue on the 10^th^ day and the 20^th^ day in a time-dependent manner, as shown in Fig. 1C and Fig. 1D, respectively. The immunocytochemistry technique was also used in order to determine the purity of HRASMCs. In brief, HRASMCs were stained with α-SM-actin, the specific monoclonal antibody, as a smooth muscle differentiation marker (Fig. 1 E; green color) and DAPI, the nuclei counterstained marker (Fig. 1F; blue color). The cell-pattern of Fig. 1G, combined with those from Fig. 1E and Fig. 1F, is almost the same as that of Fig. 1E and Fig. 1F. This clearly indicates that the cells are HRASMCs. Therefore, a single HRASMC was successfully derived from tissue from a human renal artery using the explant technique.

### The *functional* existence of a Na^+^-H^+^ exchanger (NHE)

In order to determine whether an acid-extrusion mechanism exists in the cultured HRASMCs, the experiments were first performed in HEPES-buffered superfusate (nominally free of CO_2_/HCO_3_^−^). The steady-state pH_i_ value for the cultured HRASMCs was measured as 7.19±0.03 (n = 33), in HEPES-buffered solution. The steady-state pH_i_ value of cultured HRASMCs is close to 7.2, which is the value that was reported previously for mature mammalian cells in both animal and human models [14], [16].

As shown in the left part of Fig. 2A, the pH_i_ recovers completely from intracellular acidosis that is induced using a NH_4_Cl pre-pulse technique. This result demonstrates that there is a mechanism for acid extrusion in the HRASMCs. Removal of the extracellular Na^+^ completely blocks the pH_i_ recovery from intracellular acidosis, following the NH_4_Cl pre-pulse, as shown in the middle part of Fig. 2A. The first and second columns of the histogram (Fig. 2B) show the mean pH_i_ recovery slope (measured at pH_i_  = 6.89±0.02), before and after Na^+^ removal, for six experiments. This clearly demonstrates that, under nominally CO_2_/HCO_3_^−^-free conditions, a Na^+^-dependent, but CO_2_/HCO_3_^−^-independent, acid-extrusion mechanism is involved in the pH_i_ recovery in the HRASMCs. In order to further test whether this Na^+^-dependent acid extruder is the NHE, HOE 694, a specific NHE inhibitor, was added into the superfusate. As shown in the right part of Fig. 2A, HOE 694 (30 μM) entirely inhibits the pH_i_ recovery, following the induced intracellular acidosis. The pH_i_ recovery rate (measured at pH_i_ = 6.89±0.02) for six similar experiments, which are similar to the result shown in Fig. 2A, are pooled in the first (before HOE 694 addition) and third columns (after HOE 694 addition) of Fig. 2B. These results provide clear pharmacological evidence that NHE functionally exists in cultured HRASMCs.

### The *functional* existence of a Na^+^-HCO_3_^−^ cotransporter (NBC)

The steady-state pH_i_ value of the cultured HRASMCs was measured as 7.10±0.02 (n = 24), in CO_2_/HCO_3_^−^ buffered Tyrode solution. This steady-state pH_i_ value for HRASMCs is much lower than that in HEPES-buffered Tyrode solution and the value is similar to that reported previously for mature mammalian cells, in both animal and human models [14], [16].

**Figure 3:**
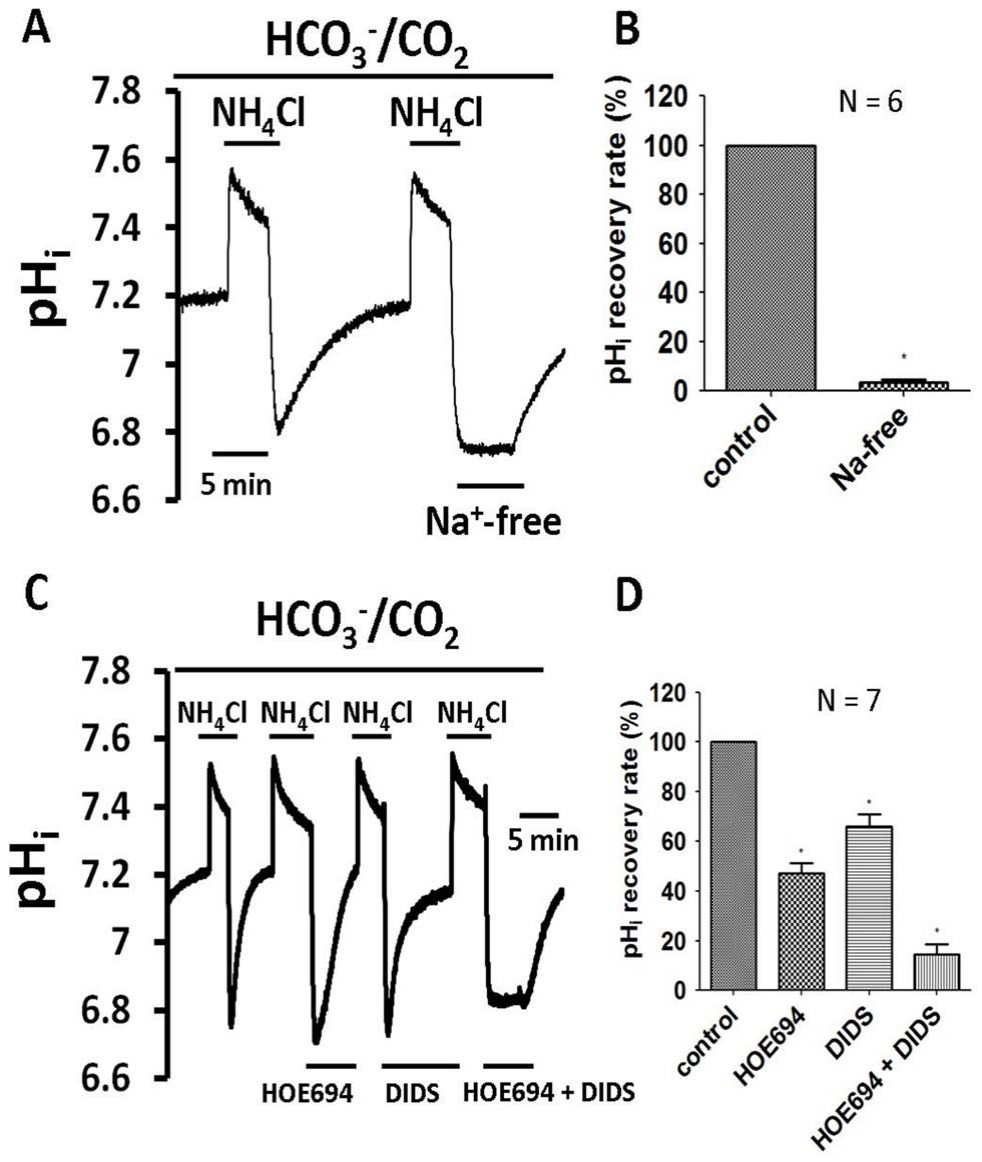
Effect of 30 μM HOE 694, Na^+^-free and 0.2 mM DIDS on pHi recovery from induced acidosis in HRASMCs superfused with 5% CO2/HCO3^−^ Tyrode solution. **A and C:** The top bar shows the buffer system used in the superfusate. The periods of application of NH_4_Cl and tested drugs (30 μM HOE 694, Na^+^-free solution, 0.2 mM DIDS and HOE 694 pulse DIDS) are shown with bars above or below the trace. The left part of traces A and C shows a typical pH_i_ recovery from an intracellular acidosis induced by a 10 min NH_4_Cl (20 mM) pre-pulse in 5% CO_2_/HCO_3_^−^ Tyrode solution (pH_o_  = 7.4, 37°C) in HRASMCs. For details of mechanism of the pre-pulse technique, please see the *Materials and Methods* section. The right part of traces A and C represents experiments showing the effect of 30 μM HOE 694 (a NHE exchanger inhibitor), Na^+^-free solution 0.2 mM DIDS (a NBC exchanger inhibitor) and HOE 694 plus DIDS on pH_i_ recovery, respectively, in HRASMCs. B and D: Histograms, showing the pH_i_ recovery slope of acid extrusion after NH_4_Cl-induced intracellular acidosis averaged for several experiments similar to those shown in A and C respectively. **: p<0.01 vs. control.

The left part of the traces shown in Figs. 3A and 3C show the pH_i_ recovery from an acid load that is induced in a CO_2_/HCO_3_^−^ environment. The removal of Na^+^ from the 5% CO_2_/HCO_3_^−^ Tyrode solution completely inhibits pH_i_ recovery, following NH_4_Cl-induced acidosis, as shown in the right part of Fig. 3A. The histogram in Fig. 3B shows the pH_i_ recovery rate, which is estimated at pH_i_ 6.83±0.03 and is the average value for six experiments in HRASMCs. This data suggests that this HCO_3_^−^-dependent acid-extrusion mechanism is also Na^+^-dependent. The pH_i_ recovery is partially blocked, as expected, in the presence of HOE 694, as shown in the second part of Fig. 3C. The second column of the histogram in Fig. 3D shows the pH_i_ recovery slope, after acid loading, for seven experiments (estimated at pH_i_ 6.81±0.08) in HRASMCs. The significant difference between the first (control) and the second column (in presence of HOE 694 in a 5% CO_2_/HCO_3_^−^ solution) shows that, apart from NHE, another HCO_3_^−^-dependent acid-extrusion mechanism is involved in the pH_i_ recovery in 5% CO_2_/HCO_3_^−^ Tyrode solution. It has been demonstrated that the stilbene drug, DIDS (0.4 mM), inhibits NBC effectively [12], [16], [25]–[28], so a further test was undertaken to determine whether DIDS inhibits this HCO_3_^−^-dependent, but HOE 694-independent, acid-extrusion mechanism, in the HRASMCs. As shown in the right part of Figs. 3C, a combination of 30 μM HOE 694 and 0.4 mM DIDS entirely inhibits the pH_i_ recovery, following induced intracellular acidosis with 5% CO_2_/HCO_3_^−^ Tyrode solution (right part of Fig. 3C), but DIDS alone does not (the third right part of Fig. 3C). The third and fourth histograms in Fig. 3D show the pH_i_ recovery rate, as an average for 7 experiments, which is similar to that shown in Fig. 3C (estimated at pH_i_ 6.80±0.08). This data demonstrates that this Na^+^- and HCO_3_^−^-dependent acid-extrusion mechanism relies on the NBC. In other words, this is the first *functional* evidence that both NHE and NBC play an important role in pH_i_ regulation through acid extrusion in cultured HRASMCs.

### The effect of LPS on the protein expression of NHE, NBC and on intracellular resting pH

**Figure 4:**
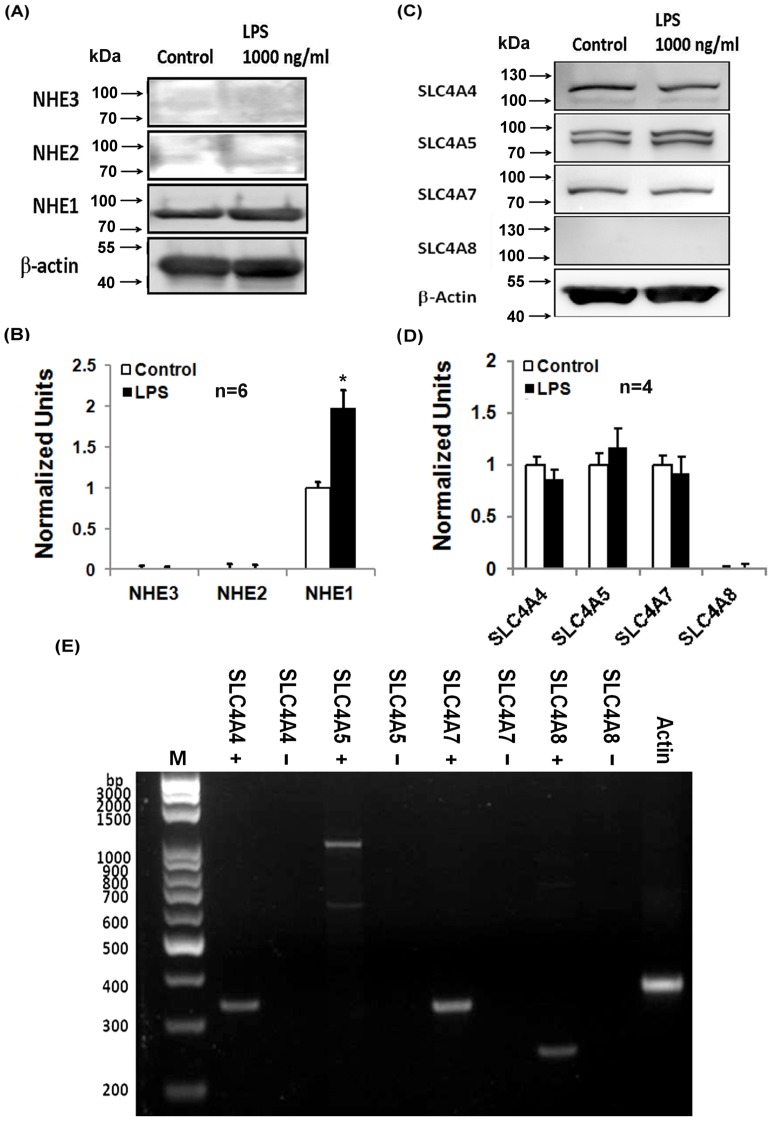
The effect of LPS on protein expression of NHE, NBC and intracellular resting pH in HRASMCs. **A**: The figure shows the result of Western blot analysis for β-actin, NHE 1, 2 and 3, from the bottom to the top, respectively, before (left part) and after (right part) the 1000 ng/ml LPS treatment (n = 4). **B**: The histogram shows relative protein expression, as an average of 6 experiments, which is similar to that shown in A. Data is shown as the mean ± SEM (p<0.01; n = 6). **C**: The figure shows the result of Western blot analysis for β-actin, SLC4A8 (NBCBE), SLC4A7 (NBCn1), SLC4A5 (NBCe2) and SLC4A4 (NBCe1), from the bottom to the top, respectively, before (left part) and after (right part) the 1000 ng/ml LPS treatment (n = 4). **D**: The histogram shows the protein expression, as an average of 4 experiments, which is similar to that shown in C. Data is shown as the mean ± SEM (p<0.01; n = 4). **E**: Gene expression of mRNA of different members of SLC4 family: NBCe1 (SLC4A4; 336 bp), NBCe2 (SLC4A5; 650 bp and 1 kb), NBCn1 (SLC4A7; 328 bp) and NDCBE1 (SLC4A8; 243 bp) extracted from HRASMCs by RT-PCR. Actin expression was used as control (373 bp). bp denotes base pairs; M denotes marker; + denotes the presence of template; − denotes the absence of template (negative control).

In order to further identify the isoform(s) of the functional NHE and NBC observed previously, the Western blot technique (see *Materials and Methods* for details) was used to test the response for treating a mouse antihuman NHE1∼3 antibody and the SLC4 family of HCO_3_^−^ transporters: SLC4A4 (NBCe1), SLC4A5 (NBCe2), SLC4A7(NBCn1) and SLC4A8 (NDCBE), respectively, in cultured HRASMCs. As shown in the left part of Fig. 4A, the isoform of NHE is purely NHE1 isoform in HRASMCs.

It is also significant that three Na^+^-coupled HCO_3_^−^ co-transporters: NBCn1 (SLC4A7; electroneutral), NBCe1 (SLC4A4; electrogenic) and NBCe2 (SLC4A5), co-exist in the cultured HRASMCs, as shown in the left part of Fig. 4C. This is different to the case for animal models, because only NBCn1 (SLC4A7) mediates the Na^+^-dependent bicarbonate transport that is important for acid extrusion in the smooth muscle cells of mice mesenteric, coronary and cerebral small arteries [12], [15], [17], [27], [31].

In order to determine whether the LPS affects NHE1 and NBCs activity, 1000 ng/ml LPS was added into cultured HRASMCs for 24 hrs. It was found that LPS (1000 ng/ml) significantly increases NHE1 protein expression (+95% ±21; n = 4, p<0.05), as shown in the right part of Fig. 4A. The histogram in Fig. 4B shows the protein expression, as an average of 6 experiments, which is similar to that shown in Fig. 4A. However, the addition of 1000 ng/ml LPS into HRASMCs for 24 hrs does not affect the protein expression in all 3 SLC4 members of NBC, i.e. NBCn1, NBCe1 and NBCe2 (n = 4, p>0.05), as shown in the right part of Fig. 4C. The histogram in Fig. 4D shows the protein expression, as an average for 4 experiments, which is similar to that shown in Fig. 4C.

Following the discovery of 3 different NBC isoforms in cultured HRASMCs, the molecular identity of the transporter responsible was determined, using the reverse transcription – polymerase chain reaction (RT-PCR) technique. As shown in Fig. 4E, the total human RNA from the primary cell was reverse transcribed and subjected to PCRs and gel electrophoresis, in order to determine the expression pattern of different SLC4 family members. It is seen that the mRNA of NBCe1 (SLC4A4), NBCe2 (SLC4A5), NBCn1 (SLC4A7) and NDCBE1 (SLC4A8) are clearly expressed, as shown in Fig. 4E. Note that the protein of NDCBE1 (SLC4A8) was not detected (Fig. 4C), while that of the mRNA level was detected (Fig. 4E). Whether this difference in protein and mRNA in NDCBE1 is caused by the less specificity of antibody or a scarcity of protein requires further study.

**Figure 5:**
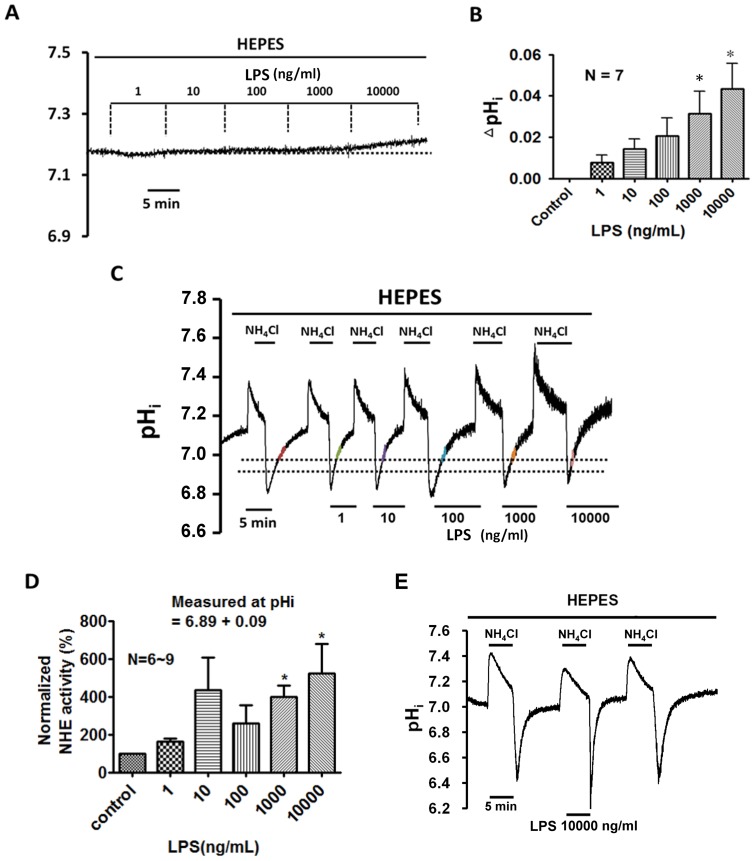
Effect of lipopolysaccharides (LPS) on resting pH_i_ and NHE activity in HRASMCs superfused with HEPES-buffered Tyrode solution. **A, C, E:** The top bar shows the buffer system used in the superfusate. The periods of application of NH_4_Cl and LPS (1∼10000 ng/ml) are shown with bars above or below the trace. Traces A represents experiments showing the effect of different concentrations of LPS (1∼10000 ng/ml) on resting pH_i_ in HEPES-buffered Tyrode solution in HRASMCs (pH_o_  = 7.4, 37°C). The left part of traces C and E shows a typical pH_i_ recovery from an intracellular acidosis induced by a 7 min NH_4_Cl (20 mM) pre-pulse in HEPES-buffered solution (pH_o_  = 7.4, 37°C) in HRASMCs. The right part of traces C and E represents experiment showing the effect of different concentrations of LPS (1∼10000 ng/ml) on pH_i_ recovery in HRASMCs. B, D: Histograms, showing the change in resting pH_i_ and pH_i_ recovery slope of acid extrusion after NH_4_Cl-induced intracellular acidosis averaged for 7 and 6 experiments similar to those shown in A and C (measured at the range between the two dash lines of the figure), respectively. *: p<0.01 vs. control.

The significant effect of superfusion with LPS (1–10000 ng/ml) on the pH_i_ of the HRASMCs is shown in Fig. 5A. In a HEPES superfusate (Fig. 5A), LPS treatment results in dose-dependent changes in the pH_i_, i.e. there is no change at lower doses (1–100 ng/ml), but there is a significant intracellular alkalosis for each 0.03 and 0.04 increase in the pH unit at higher doses of LPS, at 1000 ng/ml and 10000 ng/ml, respectively (p<0.05, n = 7). Note that the significant intracellular alkalosis (∼ +0.04 pH unit) induced by 10000 ng/ml LPS is irreversible after washout for 30 min. The histogram in Fig. 5B shows the mean LPS-induced pH_i_ changes for seven experiments, which is similar to that shown in Fig. 5A. The result clearly shows that the LPS-induced intracellular alkalosis is concentration-dependent between 1 and 10000 ng/ml, in HEPES-buffered superfusate.

### The acute and chronic effects of LPS on the Na^+^-H^+^ exchanger activity and cellular growth

As shown in the left part of Fig. 5C, the pH_i_ recovers completely from an intracellular acidosis due to the NHE in the control. As expected, LPS causes an increase in the pH_i_ recovery slope in a concentration-dependent manner, as illustrated in the right part of Fig. 5C. At higher concentrations of 1000 and 10000 ng/ml, LPS results in a four and five fold increase in the NHE activity (p<0.05; n = 6), respectively, as shown in the rightmost part of Fig. 5C. In other words, this study demonstrates that the increase in the resting pH_i_ induced by 1000 and 10000 ng/ml LPS is mainly due to its effect of increasing NHE activity. The histogram (Fig. 5D) shows the mean pH_i_ recovery slope (measured at pH_i_  = 6.89±0.09), before and after the addition of LPS, for the six experiments, which is similar to the slope shown in Fig. 5C.

In order to rule out any possible interference caused by multiple NH_4_Cl pre-pulses in the pH_i_ recovery shown in Fig. 5C, experiments were performed using less NH_4_Cl pre-pulses to determine the effect of LPS on NHE activity. As shown in the Fig. 5E, 10000 ng/ml LPS increases pH_i_ recovery, following induced acidosis (+346±56%; n = 3) that is similar to that for 10000 ng/ml in the multiple NH_4_Cl experiments shown in Fig. 5C. These LPS induced changes in pH_i_ recovery are completely reversed after washout of LPS, as shown in the far right part of Fig. 5E. This result clearly rules out any possible interference caused by multiple NH_4_Cl pre-pulses on the effect of LPS on NHE activity that is seen in the experiment in Fig. 5C.

### The effect of LPS on NBC activity

**Figure 6:**
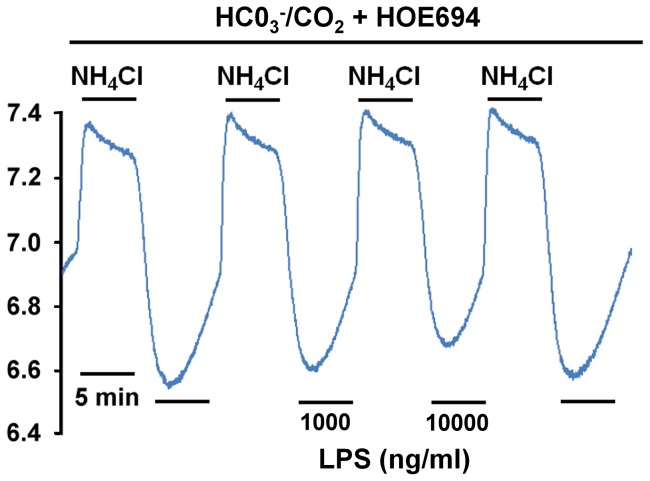
Effect of lipopolysaccharides (LPS) on NBC activity in HRASMCs superfused with 5% CO2/HCO3^−^ Tyrode solution plus 30 μM HOE 694. The top bar shows the buffer system used in the superfusate. The periods of application of NH_4_Cl and LPS (1000 and 10000 ng/ml) are shown with bars above or below the trace. The left part of traces shows a typical pH_i_ recovery from an intracellular acidosis induced by a NH_4_Cl (20 mM) pre-pulse in 5% CO_2_/HCO_3_^−^ Tyrode solution plus 30 μM HOE 694 (pH_o_  = 7.4, 37°C) in HRASMCs. The middle part of trace represents experiment showing the effect of 2 different concentrations of LPS (1000 ng/ml and 10000 ng/ml) on pH_i_ recovery in HRASMCs.

Although the result of Western blot analysis (Fig. 4C and 4D) indicates clearly that LPS has no effect on NBCs protein expression, the possible effect of LPS on NBC activity is of interest, so experiments were performed using the superfusate of 5% CO_2_/HCO_3_^−^ Tyrode solution in the presence of 30 μM HOE 694. Under these conditions, LPS treatment causes no change in NBC activity at higher doses (1000 and 10000 ng/ml), as shown in the middle part of Fig. 6 (p>0.05, n = 4). Note that the LPS-induced effect on NBC activity is totally different to that of LPS on NHE activity (Fig. 5C and Fig. 5E, respectively).

### The chronic effects of LPS on the Na^+^-H^+^ exchanger activity and cellular growth

**Figure 7:**
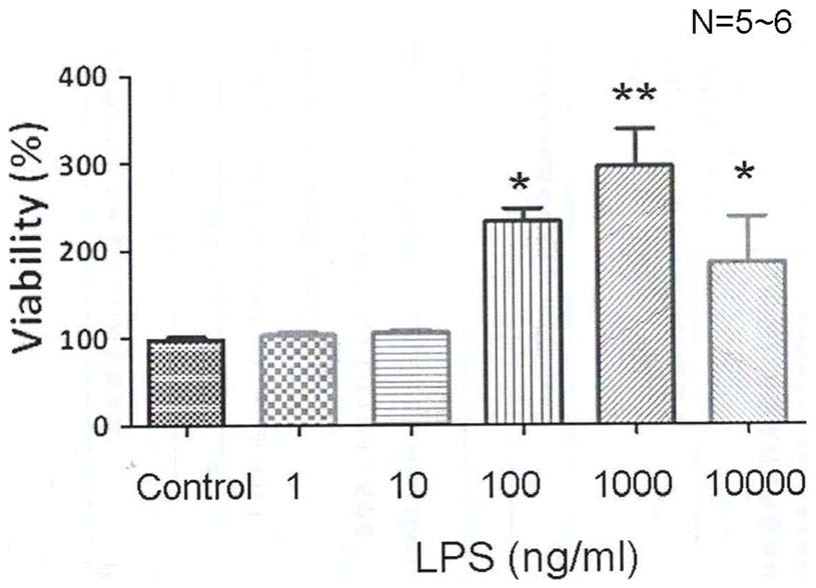
Viability effect of lipopolysaccharides (LPS) in different concentrations in HRASMC. HRASMC were incubated with different concentration of LPS (1∼10000 ng/ml) for 24 hr. Supernatants for MTT measurement were taken at 24 hr before and after the LPS challenge. Data were mean ± standard error (n = 5∼6). *p<0.05 vs. control, **p<0.001 vs. control.

The effect of the concentration of LPS (1∼10000 ng/ml) on cellular growth of cultured HRASMCs is of interest. The HRASMCs were treated with LPS for 24 hrs in a culture chamber, and then a MTT assay was used to determine the cell viability (growth). It was found that LPS increases the growth of cultured HRASMCs in a concentration-dependent manner (Fig. 7), i.e., it has no effect between 1∼10 ng/ml, while cell growth increased ∼2 fold or ∼3 fold, respectively, at higher dosages of 100 ng/ml and 1000 ng/ml (p<0.05, n = 6). Note that the highest dosage of 10000 ng/ml LPS does increase the number of cells ∼1.9 fold (p<0.05, n = 5), which is not greater than that the effect of 100 and 1000 ng/ml. These results are the first solid evidence that LPS increases cellular growth in cultured HRASMCs.

**Figure 8:**
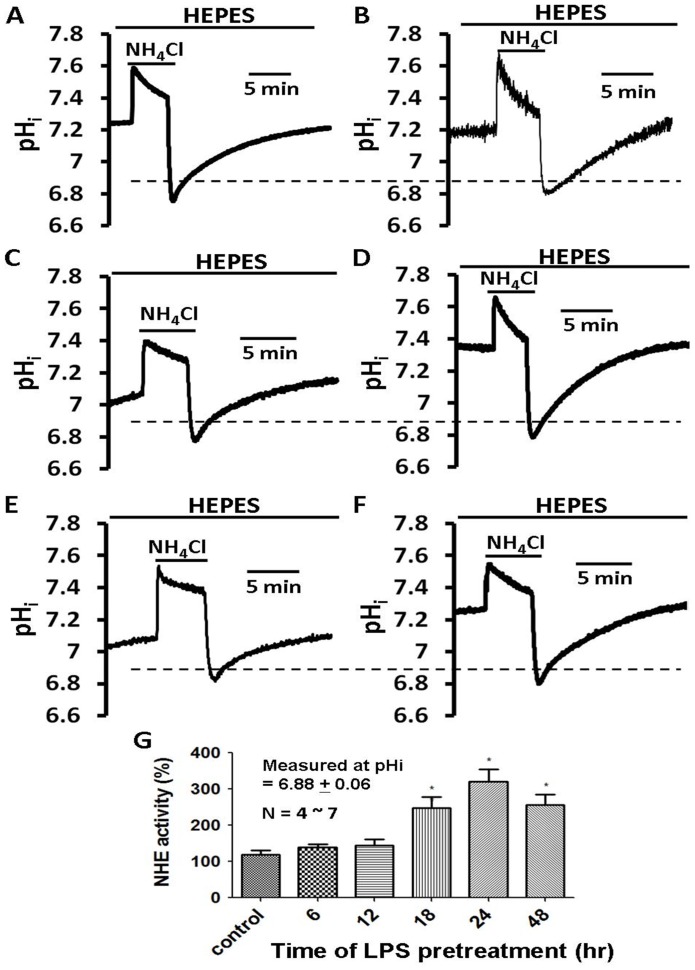
Time-dependent effect of lipopolysaccharides (LPS) on NHE activity in HRASMCs. **A∼F**: HRASMCs were incubated with different LPS (1000 ng/ml) for 0, 6, 12, 18, 24 and 48 hr, respectively. Cells were subjects to perform NH_4_Cl pre-pulse method to detect the NHE activity. Top bar shows the buffer system used in the superfusate. The periods of application of NH_4_Cl and tested LPS (1000 ng/ml) are shown by the bars below or above the traces. Traces A shows a typical pH_i_ recovery from an intracellular acidosis induced by a 10 min NH_4_Cl (20 mM) pre-pulse in HEPES-buffered solution (pH_o_  = 7.4, 37°C) in HRASMCs. Traces B∼F represent experiments showing the time-dependent effect of LPS (6, 12, 18, 24 and 48 hr, respectively) on NHE activity in HRASMCs. **G**: Histogram, shows acid extrusion after acid loading estimated at pH_i_ 6.88±0.06, averaged for several experiments similar like that of A∼F, respectively. **p<0.01 vs. control.

In order to verify whether the phenomenon of LPS-induced cellular growth is closely related to NHE activity, the time-dependent effect of 1000 ng/ml LPS on the NHE activity was determined, as shown in Fig. 8. The choice of a 1000 ng/ml dose is based on the result of its significant effect on NHE1 protein synthesis (Fig. 4A), NHE activity and pH_i_ (Fig. 5). The NHE activity was observed before and after the addition of LPS (1000 ng/ml), at 6, 12, 18, 24 and 48 hrs, in the culture chamber, as shown in Fig. 8A – Fig. 8F, respectively. This study finds that NHE activity is significantly increased at 18 hr ∼48 hr (Fig. 8D ∼ Fig. 8F, respectively), but not significantly increased before 12 hr (Fig. 8B, Fig. 8C). The histograms in Fig. 8G show the normalized NHE activity (measured at pH_i_  = 6.88±0.06), which is similar to that shown in Figs. 8A ∼8F, respectively. Note that the maximum increase due to the 1000 ng/ml dose is observed at 24 hr (Fig. 8G).

These results provided clear pharmacological evidence that, in HEPES-buffered Tyrode solution, the underlying mechanism for LPS-induced, concentration-dependent intracellular alkalosis (Fig. 5A) in cultured HRASMCs is mainly due to its effect on NHE expression/activity (Fig. 4, Fig. 5 and Fig. 8). This is the first demonstration that the concentration-dependent LPS-induced cellular growth of culture HRASMCs (Fig. 7) is possibly due to the pH_i_ change that is caused by an increase in NHE activity (Fig. 5 and Fig. 8).

## Discussion

### The evidence of acid extruding regulators (NHE1, NBCn1, NBCe1 and NBCe2)

Using microspectrofluorimetry, this study provides the first straightforward and convincing pharmacological evidence that NHE1 and another three HOC_3_^−^-dependent acid extruder, i.e. NBC, are functionally responsible for acid extrusion, following induced acidosis in human renal artery smooth muscle. NHE's activity is HCO_3_^−^-independent and Na^+^ dependent (Fig. 2) [16], [26], [47]. This conclusion is confirmed by the finding that the acid extruder is entirely blocked by HOE 694 (Fig. 2), which is a highly-specific NHE-1 inhibitor [21]. Molecular biological analysis shows that, of the nine different members of NHE, i.e. NHE 1∼9 [22], the NHE1 protein is identified as the protein that ubiquitously expresses in different tissues, including heart and smooth muscle [48], [49]. It is shown that HOE 694 exhibits a high selectivity for cloned & expressed NHE1 that is two or more orders of magnitude higher than for the other isoforms, such as NHE 2 & 3 [50]. These results show that the functioning NHE in the HRASMCs is also sensitive to low concentration of HOE 694 (30 μM) (Fig. 2). Western blot analysis also provides straightforward evidence that the NHE isoform is purely NHE1 (Fig. 4A) and lacks NHE 2 and NHE 3. Whether this data excludes a significant presence for other members of NHE (4∼9) in HRASMCs is worthy of discussion. It appears that it is excluded, on the grounds that the data available for NHE 4, 5 indicates that the acid extruder is essentially relatively insensitive to HOE694, and NHE 6∼9 only exists in the membrane of intracellular organelles [22]. Therefore, using pharmacological methods and a molecular probe, this study provides direct evidence that the native NHE that functions during pH_i_-regulation in the HRASMCs is the NHE-1 isoform; not other members of the NHE proteins.

Another category extruding mechanism whose activity is HCO_3_^−^- and Na^+^-dependent (Fig. 3A) is NBC. This is supported by another result (Fig. 3C) in this study, which shows that NBC is sensitive to DIDS, a NBC inhibitor, and insensitive to HOE 694 [16], [21], [28], [31], [47]. Relevant molecular candidates for Na^+^-dependent bicarbonate transport include at least five members of the slc4 family, including 2 electrogenic Na^+^, HCO_3_^−^ cotransporters (NBCe1/SLC4A4 and NBCe2/SLC4A5), 1 electroneutral Na^+^, HCO_3_^−^ cotransporter (NBCn1/SLC4A7) and 2 Na^+^-dependent Cl^−^/HCO_3_^−^ exchangers (NCBE/SLC4A10 and NDCBE/SLC4A8) [12], [30], [33]. Recently, both in rat and mouse smooth muscle cells, the Aalkjaer group demonstrated that the NBC is NBCn1, i.e. it is electroneutral [12], [15], [31], [44]. They also found that disruption of the Na^+^, HCO_3_^−^-cotransporter NBCn1 (SLC4A7) inhibits NO- mediated vasorelaxation, smooth muscle Ca^2+^-sensitivity and the development of hypertension in mice [17]. Indeed, this study demonstrates functionally that a Na^+^ and HCO_3_^−^ dependent acid-extruding mechanism is responsible for acid extrusion in the cultured HRASMCs (Fig. 3A). Surprisingly, this study demonstrates, for the first time, that three different isoforms of NBC: NBCn1 (SLC4A7; electroneutral), NBCe1 (SLC4A4; electrogenic) and NBCe2 (SLC4A5), are detected in the protein/mRNA level (see Fig. 5B and Fig. 5E) in the cultured HRASMCs. In other words, the co-existence of 3 types NBC in this study is different to that found in mouse and rat models (c.f. Aalkjaer's group), which is probably due to differences in species/organs, if the specificity of the antibodies used is reliable (see *Materials and Methods* for details). Moreover, it is important to consider another possibility. The observed isoforms of NBC may be directly caused by the altered expression of the protein, during the culture in question, or a secondary, for instance the compensatory up- or down-regulation of other proteins, as cell culture is known to alter the expression profile [44]. Knowledge of the exact stoichiometry between HCO_3_^−^ and Na^+^ (coupling ratio) and the extent to which the NBC is electrogenic or electroneutral to the multiple NBC isoforms in the cultured HRASMCs will involve further study.

### The potential role of inhibitors of NHE1 and NBCs in a clinic

In the HRASMCs, it is demonstrated that the activity of NHE1 and/or NBCs (n1, e1 and e2) is imperative for pH_i_ regulation (Fig. 2, Fig. 3 and Fig. 4). Both in rat or mouse vascular smooth cells, it is seen that NHE1 is predominantly active at lower pH_i_ values and that it plays a major role in acid extrusion under conditions of severe intracellular acidification [18], but NBCn1 is active at both low and near-physiological pH_i_ values [15], [17]. Whether the percentage contributions of NHE1 and NBCs of HRASMCs are similar to that of those for rat or mouse vascular smooth cells will be a subject for further study. In order to quantify the pH_i_-dependency of NHE and NBC activity, an entire set of experiments to elaborately check the intracellular total buffering power (βtot), which comprises the intrinsic- and CO_2_-related buffering power, must firstly occur [14].

According to this result, it is predicted that the alteration of the activity of NHE1 and NBC plays a vital role in maintaining many physiological functions, such as cell differentiation, growth and apoptosis in HRASMCs, which is similar to that found for many other groups in other cell types [10], [11], [43], [51]. It is also clear that NHE and NBCs may provide an important method for the prevention of some acute and chronic, pathological vascular illnesses in clinics, such as ischaemia-reperfusion induced damage that is caused by rapid recovery of pH_i_ [52] and ARAS-induced atherosclerotic renovascular disease [3], [4], [12], [53], [54]. This study also implies that, as well as increasing the knowledge of the basic physiological mechanism of NBCs, the development of a new and specific NBCs inhibitor is another step in the process to prevent ischaemia/reperfusion-induced cardiovascular injury.

### Clinical implication of concentration- and time- dependent effects of LPS on pH_i_, NHE and NBCs

There is a close relationship between the plasma concentration of circulating LPS and the development of multiple organ failure and death in patients with bacteriologically verified systemic meningococcal disease (SMD) [55]. A plasma LPS level of more than 700 ng/L is correlated with the development of severe septic shock (P<0.0001), adult respiratory distress syndrome (P = 0.0035), a pathologically elevated serum creatinine level (P<0.0001), or death, as a consequence of multiple organ failure (P = 0.0002). Initial plasma LPS levels of less than 25, 25–700, 700–10000 and greater than 10000 ng/L are also associated with a 0%, 14%, 27% and 86% risk of fatality, respectively [55]. Indeed, recently, it has been demonstrated that mRNA and protein expression of toll-like receptor 4 (TLR4) are up-regulated by LPS in human aortic smooth muscles, in a dose- (10∼1000 ng/ml) and time-dependent (0–48 hr) manner [33]. In human arterial smooth muscle, LPS (10 ng/ml) has also been found to induce mRNA and protein expression of matrix metalloproteinases-9 (MMP-9) and the process depends on TLR4/NF-kB [34]. This study, determines the effect of LPS at different concentrations (1∼10000 ng/ml) LPS (Figs. 4–8). This is the first evidence that LPS (1∼10000 ng/ml) induces concentration-dependent, intracellular alkalosis (Fig. 5A). Fig. 5C and Fig. 8 show that the LPS increases NHE activity in both a concentration- and time-dependent manner. However, LPS has no effect on either the protein expression or activity of NBCs (n1, e1 and e2), the other main acid-extruding mechanism in HRASMCs (Fig. 4C and Fig. 6). Therefore, it is demonstrated that, in HRASMCs, the LPS-induced intracellular alkalosis is mainly due to the alternation of NHE1 activity/protein expression.

As well as from cell differentiation, growth and apoptosis are sensitive to changes in pH_i_ [10], [11]. It has been claimed that irreversible endothelial dysfunction and vascular atherosclerosis are related to a disturbance in pH_i_ [12], [13]. For example, NHE1 activity has been proven to play a vital role in proliferation, both in carcinogenic and non-carcinogenic cells [56]–[58]. Recently, it has been found that LPS-induced vascular inflammation/occlusion and systemic organ failure are initially triggered by vascular the endothelial apoptosis that is associated with the activating calpain, which is a calcium-dependent protease, and increased [Ca^2+^]_i_ [59]. A very recently study has also demonstrated that treatment of HUVECs with LPS increases NHE1 activity in a time-dependent manner that is associated with increased [Ca^2+^]_i_, which results in enhanced calpain activity and in HUVECs apoptosis, via NHE1-dependent degradation of Bcl-2, which is one of the anti-apoptotic family members [38]. Indeed, this study also shows, for the first time, that LPS increases cellular growth significantly in a concentration-dependent manner (Fig. 7). The pattern of change of LPS-induced cell growth is also closely related to those of LPS-induced changes in pH_i_ and increases in NHE activity (Fig. 5, Fig. 7 and Fig. 8).

LPS is one of the main inflammatory mediators that exerts various atherogenic effects that involve the expression of adhesion molecules [25], [60]. For example, LPS has been demonstrated to stimulate the release of INF-γ, IL (interlukin) -1, IL-6, IL-8, TNF-α (tumor necrosis factor-alfa) and GM-CSF (granulocyte-macrophage colony-stimulating factor), either through direct or indirect mechanisms [25], [60]. If a change in NHE1 activity also affects LPS-induced impact on cytokines, then the development of specific and potent NHE inhibitors/agonists may produce a cure for LPS induced-malfunction, such as organ failures in sepsis and septic shock, in a clinic. Therefore, determining whether these LPS-induced alternations to cytokines, peptides and co-stimulatory molecules is related to changes in pH_i_ and NHE activity is worthy of future study.

## References

[1] Jacobson HR (1988) Ischemic renal disease: an overlooked clinical entity? Kidney Int 34: 729–743. 3059028 10.1038/ki.1988.240

[2] Zoccali C, Mallamaci F, Finocchiaro P (2002) Atherosclerotic renal artery stenosis: epidemiology, cardiovascular outcomes, and clinical prediction rules. J Am Soc Nephrol 13: S179–183. 12466310 10.1097/01.asn.0000032548.18973.0f

[3] Vashist A, Heller EN, Brown EJ Jr, Alhaddad IA (2002) Renal artery stenosis: a cardiovascular perspective. Am Heart J 143: 559–564. 11923791 10.1067/mhj.2002.120769

[4] Manjunath G, Tighiouart H, Ibrahim H, MacLeod B, Salem DN, et al (2003) Level of kidney function as a risk factor for atherosclerotic cardiovascular outcomes in the community. J Am Coll Cardiol 41: 47–55. 12570944 10.1016/s0735-1097(02)02663-3

[5] Conlon PJ, Little MA, Pieper K, Mark DB (2001) Severity of renal vascular disease predicts mortality in patients undergoing coronary angiography. Kidney Int 60: 1490–1497. 11576364 10.1046/j.1523-1755.2001.00953.x

[6] Pillay WR, Kan YM, Crinnion JN, Wolfe JH (2002) Prospective multicentre study of the natural history of atherosclerotic renal artery stenosis in patients with peripheral vascular disease. Br J Surg 89: 737–740. 12027983 10.1046/j.1365-2168.2002.02144.x

[7] Grinstein S, Woodside M, Sardet C, Pouyssegur J, Rotin D (1992) Activation of the Na^+^/H^+^ antiporter during cell volume regulation. Evidence for a phosphorylation-independent mechanism. J Biol Chem 267: 23823–23828. 1331102

[8] Kiss L, Korn SJ (1999) Modulation of N-type Ca^2+^ channels by intracellular pH in chick sensory neurons. J Neurophysiol 81: 1839–1847. 10200218 10.1152/jn.1999.81.4.1839

[9] Jeremy RW, Koretsune Y, Marban E, Becker LC (1992) Relation between glycolysis and calcium homeostasis in postischemic myocardium. Circ Res 70: 1180–1190. 1576739 10.1161/01.res.70.6.1180

[10] Grinstein S, Rotin D, Mason MJ (1989) Na^+^/H^+^ exchange and growth factor-induced cytosolic pH changes. Role in cellular proliferation. Biochim Biophys Acta 988: 73–97. 2535787 10.1016/0304-4157(89)90004-x

[11] Goossens JF, Henichart JP, Dassonneville L, Facompre M, Bailly C (2000) Relation between intracellular acidification and camptothecin-induced apoptosis in leukemia cells. Eur J Pharm Sci 10: 125–131. 10727878 10.1016/s0928-0987(99)00091-3

[12] Boedtkjer E, Aalkjaer C (2013) Acid-base transporters modulate cell migration, growth and proliferation: Implications for structure development and remodeling of resistance arteries? Trends Cardiovasc Med 23: 59–65. 23266155 10.1016/j.tcm.2012.09.001

[13] Son SM, Whalin MK, Harrison DG, Taylor WR, Griendling KK (2004) Oxidative stress and diabetic vascular complications. Curr Diab Rep 4: 247–252. 15265465 10.1007/s11892-004-0075-8

[14] Leem CH, Lagadic-Gossmann D, Vaughan-Jones RD (1999) Characterization of intracellular pH regulation in the guinea-pig ventricular myocyte. J Physiol 517: 159–180. 10226157 10.1111/j.1469-7793.1999.0159z.x PMC2269328

[15] Boedtkjer E, Praetorius J, Aalkjaer C (2006) NBCn1 (slc4a7) mediates the Na^+^-dependent bicarbonate transport important for regulation of intracellular pH in mouse vascular smooth muscle cells. Circ Res 98: 515–523. 16439691 10.1161/01.RES.0000204750.04971.76

[16] Loh SH, Chen WH, Chiang CH, Tsai CS, Lee GC, et al (2002) Intracellular pH regulatory mechanism in human atrial myocardium: functional evidence for Na^+^/H^+^ exchanger and Na^+/^HCO_3_ ^−^ symporter. J Biomed Sci 9: 198–205. 12065894 10.1007/BF02256066

[17] Boedtkjer E, Praetorius J, Matchkov VV, Stankevicius E, Mogensen S, et al (2011) Disruption of Na^+^, HCO_3_ ^−^ cotransporter NBCn1 (slc4a7) inhibits NO-mediated vasorelaxation, smooth muscle Ca^2+^ sensitivity, and hypertension development in mice. Circulation 124: 1819–1829. 21947296 10.1161/CIRCULATIONAHA.110.015974

[18] Boedtkjer E, Damkier HH, Aalkjaer C (2012) NHE1 knockout reduces blood pressure and arterial media/lumen ratio with no effect on resting pH_i_ in the vascular wall. J Physiol 590: 1895–1906. 22351634 10.1113/jphysiol.2011.227132 PMC3573311

[19] Aronson PS (1985) Kinetic properties of the plasma membrane Na^+^-H^+^ exchanger. Annu Rev Physiol 47: 545–560. 2581505 10.1146/annurev.ph.47.030185.002553

[20] Grinstein S, Rothstein A (1986) Mechanisms of regulation of the Na^+^/H^+^ exchanger. J Membr Biol 90: 1–12. 3009822 10.1007/BF01869680

[21] Loh SH, Sun B, Vaughan-Jones RD (1996) Effect of Hoe 694, a novel Na^+^-H^+^ exchange inhibitor, on intracellular pH regulation in the guinea-pig ventricular myocyte. Br J Pharmacol 118: 1905–1912. 8864522 10.1111/j.1476-5381.1996.tb15623.x PMC1909868

[22] Bobulescu IA, Di Sole F, Moe OW (2005) Na^+^/H^+^ exchangers: physiology and link to hypertension and organ ischemia. Curr Opin Nephrol Hypertens 14: 485–494. 16046909 10.1097/01.mnh.0000174146.52915.5d PMC2861558

[23] Goyal S, Vanden Heuvel G, Aronson PS (2003) Renal expression of novel Na^+^/H^+^ exchanger isoform NHE8. Am J Physiol Renal Physiol 284: F467–473. 12409279 10.1152/ajprenal.00352.2002

[24] Kalaria RN, Premkumar DR, Lin CW, Kroon SN, Bae JY, et al (1998) Identification and expression of the Na^+^/H^+^ exchanger in mammalian cerebrovascular and choroidal tissues: characterization by amiloride-sensitive [3H]MIA binding and RT-PCR analysis. Brain Res Mol Brain Res 58: 178–187. 9685633 10.1016/s0169-328x(98)00108-9

[25] Chow JC, Young DW, Golenbock DT, Christ WJ, Gusovsky F (1999) Toll-like receptor-4 mediates lipopolysaccharide-induced signal transduction. J Biol Chem 274: 10689–10692. 10196138 10.1074/jbc.274.16.10689

[26] Loh SH, Jin JS, Tsai CS, Chao CM, Chiung CS, et al (2002) Functional evidence for intracellular acid extruders in human ventricular myocardium. Jpn J Physiol 52: 277–284. 12230804 10.2170/jjphysiol.52.277

[27] Romero MF, Chen AP, Parker MD, Boron WF (2013) The SLC4 family of bicarbonate (HCO_3_ ^−^) transporters. Mol Aspects Med 34: 159–182. 23506864 10.1016/j.mam.2012.10.008 PMC3605756

[28] Romero MF, Hediger MA, Boulpaep EL, Boron WF (1997) Expression cloning and characterization of a renal electrogenic Na^+^/HCO_3_ ^−^ cotransporter. Nature 387: 409–413. 9163427 10.1038/387409a0

[29] Lagadic-Gossmann D, Buckler KJ, Vaughan-Jones RD (1992) Role of bicarbonate in pH recovery from intracellular acidosis in the guinea-pig ventricular myocyte. J Physiol 458: 361–384. 1302269 10.1113/jphysiol.1992.sp019422 PMC1175160

[30] Romero MF, Fulton CM, Boron WF (2004) The SLC4 family of HCO_3_ ^−^ transporters. Pflugers Arch 447: 495–509. 14722772 10.1007/s00424-003-1180-2

[31] Thomsen AB, Kim S, Aalbaek F, Aalkjaer C, Boedtkjer E (2014) Intracellular acidification alters myogenic responsiveness and vasomotion of mouse middle cerebral arteries. J Cereb Blood Flow Metab 34: 161–8. 24192638 10.1038/jcbfm.2013.192 PMC3887363

[32] Morrison DC, Ryan JL (1987) Endotoxins and disease mechanisms. Annu Rev Med 38: 417–432. 3555304 10.1146/annurev.me.38.020187.002221

[33] Li H, He Y, Zhang J, Sun S, Sun B (2007) Lipopolysaccharide regulates toll-like receptor 4 expression in human aortic smooth muscle cells. Cell Biol Int 31: 831–835. 17344072 10.1016/j.cellbi.2007.01.034

[34] Li H, Xu H, Sun B (2012) Lipopolysaccharide regulates MMP-9 expression through TLR4/NF-kappaB signaling in human arterial smooth muscle cells. Mol Med Rep 6: 774–778. 22842850 10.3892/mmr.2012.1010

[35] Schelling JR, Abu Jawdeh BG (2008) Regulation of cell survival by Na^+^/H^+^ exchanger-1. Am J Physiol Renal Physiol 295: F625–632. 18480176 10.1152/ajprenal.90212.2008 PMC2653110

[36] Garciarena CD, Caldiz CI, Portiansky EL, Chiappe de Cingolani GE, Ennis IL (2009) Chronic NHE-1 blockade induces an antiapoptotic effect in the hypertrophied heart. J Appl Physiol 106: 1325–1331. 19179646 10.1152/japplphysiol.91300.2008

[37] Wang HL, Akinci IO, Baker CM, Urich D, Bellmeyer A, et al (2007) The intrinsic apoptotic pathway is required for lipopolysaccharide-induced lung endothelial cell death. J Immunol 179: 1834–1841. 17641050 10.4049/jimmunol.179.3.1834

[38] Zhao Y, Cui G, Zhang N, Liu Z, Sun W, et al (2012) Lipopolysaccharide induces endothelial cell apoptosis via activation of Na^+^/H^+^ exchanger 1 and calpain-dependent degradation of Bcl-2. Biochem Biophys Res Commun 427: 125–132. 22995319 10.1016/j.bbrc.2012.09.023

[39] Fletcher PS, Elliott J, Grivel JC, Margolis L, Anton P, et al (2006) Ex vivo culture of human colorectal tissue for the evaluation of candidate microbicides. AIDS 20: 1237–1245. 16816551 10.1097/01.aids.0000232230.96134.80

[40] Lam TI, Wise PM, O'Donnell ME (2009) Cerebral microvascular endothelial cell Na/H exchange: evidence for the presence of NHE1 and NHE2 isoforms and regulation by arginine vasopressin. Am J Physiol Cell Physiol 297: C278–289. 19458287 10.1152/ajpcell.00093.2009 PMC2724093

[41] De Giusti VC, Orlowski A, Villa-Abrille MC, de Cingolani GE, Casey JR, et al (2011) Antibodies against the cardiac sodium/bicarbonate co-transporter (NBCe1) as pharmacological tools. Br J Pharmacol 164: 1976–1989. 21595652 10.1111/j.1476-5381.2011.01496.x PMC3246661

[42] Orlowski A, De Giusti VC, Morgan PE, Aiello EA, Alvarez BV (2012) Binding of carbonic anhydrase IX to extracellular loop 4 of the NBCe1 Na^+^/HCO_3_ ^−^ cotransporter enhances NBCe1-mediated HCO_3_ ^−^ influx in the rat heart. Am J Physiol Cell Physiol 303: C69–80. 22538240 10.1152/ajpcell.00431.2011

[43] Teshima Y, Akao M, Jones SP, Marban E (2003) Cariporide (HOE642), a selective Na^+^-H^+^ exchange inhibitor, inhibits the mitochondrial death pathway. Circulation 108: 2275–2281. 14568900 10.1161/01.CIR.0000093277.20968.C7

[44] Boedtkjer E, Aalkjaer C (2012) Intracellular pH in the resistance vasculature: regulation and functional implications. J Vasc Res 49: 479–496. 22907294 10.1159/000341235

[45] Buckler KJ, Vaughan-Jones RD, Peers C, Lagadic-Gossmann D, Nye PC (1991) Effects of extracellular pH, PCO_2_ and HCO_3_ ^−^ on intracellular pH in isolated type-I cells of the neonatal rat carotid body. J Physiol 444: 703–721. 1822566 10.1113/jphysiol.1991.sp018902 PMC1179957

[46] Roos A, Boron WF (1981) Intracellular pH. Physiol Rev 61: 296–434. 7012859 10.1152/physrev.1981.61.2.296

[47] Cingolani HE, Alvarez BV, Ennis IL, Camilion de Hurtado MC (1998) Stretch-induced alkalinization of feline papillary muscle: an autocrine-paracrine system. Circ Res 83: 775–780. 9776724 10.1161/01.res.83.8.775

[48] Fliegel L, Sardet C, Pouyssegur J, Barr A (1991) Identification of the protein and cDNA of the cardiac Na^+^/H^+^ exchanger. FEBS Lett 279: 25–29. 1704856 10.1016/0014-5793(91)80241-t

[49] Orlowski J, Kandasamy RA, Shull GE (1992) Molecular cloning of putative members of the Na/H exchanger gene family. cDNA cloning, deduced amino acid sequence, and mRNA tissue expression of the rat Na/H exchanger NHE-1 and two structurally related proteins. J Biol Chem 267: 9331–9339. 1577762

[50] Counillon L, Scholz W, Lang HJ, Pouyssegur J (1993) Pharmacological characterization of stably transfected Na^+^/H^+^ antiporter isoforms using amiloride analogs and a new inhibitor exhibiting anti-ischemic properties. Mol Pharmacol 44: 1041–1045. 8246907

[51] Sun HY, Wang NP, Halkos ME, Kerendi F, Kin H, et al (2004) Involvement of Na^+^/H^+^ exchanger in hypoxia/re-oxygenation-induced neonatal rat cardiomyocyte apoptosis. Eur J Pharmacol 486: 121–131. 14975701 10.1016/j.ejphar.2003.12.016

[52] Bond JM, Herman B, Lemasters JJ (1991) Protection by acidotic pH against anoxia/reoxygenation injury to rat neonatal cardiac myocytes. Biochem Biophys Res Commun 179: 798–803. 1898402 10.1016/0006-291x(91)91887-i

[53] Scholz W, Albus U, Lang HJ, Linz W, Martorana PA, et al (1993) Hoe 694, a new Na^+^/H^+^ exchange inhibitor and its effects in cardiac ischaemia. Br J Pharmacol 109: 562–568. 8358557 10.1111/j.1476-5381.1993.tb13607.x PMC2175687

[54] Hotta Y, Nakagawa J, Ishikawa N, Wakida Y, Ando H, et al (2001) Protective effect of SM-20550, a selective Na^+^-H^+^ exchange inhibitor, on ischemia-reperfusion-injured hearts. J Cardiovasc Pharmacol 37: 143–154. 11209997 10.1097/00005344-200102000-00002

[55] Brandtzaeg P, Kierulf P, Gaustad P, Skulberg A, Bruun JN, et al (1989) Plasma endotoxin as a predictor of multiple organ failure and death in systemic meningococcal disease. J Infect Dis 159: 195–204. 2492587 10.1093/infdis/159.2.195

[56] Putney LK, Denker SP, Barber DL (2002) The changing face of the Na^+^/H^+^ exchanger, NHE1: structure, regulation, and cellular actions. Annu Rev Pharmacol Toxicol 42: 527–552. 11807182 10.1146/annurev.pharmtox.42.092001.143801

[57] Delvaux M, Bastie MJ, Chentoufi J, Cragoe EJ Jr, Vaysse N, et al (1990) Amiloride and analogues inhibit Na^+^-H^+^ exchange and cell proliferation in AR42J pancreatic cell line. Am J Physiol 259: G842–849. 2173418 10.1152/ajpgi.1990.259.5.G842

[58] Kapus A, Grinstein S, Wasan S, Kandasamy R, Orlowski J (1994) Functional characterization of three isoforms of the Na^+^/H^+^ exchanger stably expressed in Chinese hamster ovary cells. ATP dependence, osmotic sensitivity, and role in cell proliferation. J Biol Chem 269: 23544–23552. 8089122

[59] Liu T, Huang Y, Likhotvorik RI, Keshvara L, Hoyt DG (2008) Protein Never in Mitosis Gene A Interacting-1 (PIN1) regulates degradation of inducible nitric oxide synthase in endothelial cells. Am J Physiol Cell Physiol 295: C819–827. 18650263 10.1152/ajpcell.00366.2007

[60] Akira S, Takeda K, Kaisho T (2001) Toll-like receptors: critical proteins linking innate and acquired immunity. Nat Immunol 2: 675–680. 11477402 10.1038/90609

